# Characterization of Mutton Volatile Compounds in Youzhou Dark Goats and Local White Goats Using Flavoromics, Metabolomics, and Transcriptomics

**DOI:** 10.3390/foods14234114

**Published:** 2025-12-01

**Authors:** Jie Li, Shipeng Lv, Cancan Chen, Jing Jiang, Xiaoyan Sun, Gaofu Wang, Hangxing Ren

**Affiliations:** Chongqing Academy of Animal Sciences, Rongchang 402460, China; cqhyljie@163.com (J.L.);

**Keywords:** goat meat, volatile compounds, flavoromics, untargeted metabolomics, transcriptomics

## Abstract

Native goat breeds in China are highly valued for their distinctive flavor. This study integrated flavoromics, metabolomics, and transcriptomics to analyze the flavor compounds in the meat of Youzhou Dark (WY) and Local White (BY) goats. Ten 12-month-old castrated male WY and BY goats, five each, were selected for slaughter after undergoing the same feeding and management conditions. *Longissimus dorsi* muscle were collected from each group and subjected to flavoromics, metabolomics, and transcriptomics analyses. Flavoromics identified 228 volatile compounds, of which alcohols, ketones, and esters were the most prominent. Using multivariate statistical analysis and variable importance on projection (VIP) methods, 85 differential flavor compounds between WY and BY goats were identified. The key characteristic compounds, heptanal,1-octen-3-one,2,3-butanedione, 2-methyl-butanal, and 2-pentyl-furan, effectively distinguished between the volatile profiles of the two goat breeds. Untargeted metabolomics identified a total of 47 differential metabolites with significant differences between WY and BY goats. Differences in flavor compounds between the two goat breeds were linked to the expression of genes in metabolic pathways. The genes involved in tyrosine and phenylalanine metabolism were different in the two groups of goat meat. This variation may contribute to the differences in the sensory flavor profiles of WY and BY goats. Overall, these findings provide insight into the molecular mechanisms of flavor formation in native Chinese goats and offer a foundation for improving meat quality.

## 1. Introduction

China has a long history of goat farming, with indigenous breeds offering abundant genetic resources. Goat rearing has served as a key source of income for farmers, while mutton consumption continues to rise with evolving dietary trends. Most native Chinese goat breeds are prized for their distinctive flavor profiles. Their unique flavors, combined with their tenderness, nutritional benefits, and low cholesterol content, contribute to their popularity. Flavor is a primary factor influencing consumer choice, often outweighing tenderness in ruminant meats due to its unique sensory characteristics [1]. Consumers typically assess mutton quality through vision and smell, making precise flavor evaluation challenging. Goat meat is influenced by multiple factors, including the breed, age, sex, rearing practices, slaughter methods, and processing techniques [2,3,4]. Aroma development is largely governed by volatiles, such as sulfides, aldehydes, acids, and heterocyclic compounds [5]. The complexity of flavor stems from both the intrinsic composition of the meat and the underlying genetic variations between breeds.

Recent progress in metabolomics has enabled integrative multi-omics approaches, allowing cross validation across molecular levels and the construction of gene regulatory networks to elucidate meat quality traits. Untargeted metabolomics has been used widely to determine the nature of metabolites in food, and it is effective for studying meat traits [6,7,8]. Flavoromics, recognized for its high resolution and sensitivity, effectively accurately captures a broad spectrum of volatiles and has been widely used in food research [9,10]. The flavor profiles of livestock products (meat and milk) have been well-documented, including pork [11,12], yak [13], sheep [14], chicken [15], and geese [16]. In addition, RNA sequencing (RNA-seq) has become a vital technique for analyzing the differential expression of transcriptomes in many fields, including those related to meat quality in goat [17,18]. The flavoromics approach provides a novel perspective for correlating the differential molecular expression patterns to flavor composition. Integrating multiple omics technologies is conducive to comprehensive research on meat quality and flavor formation [19].

Youzhou Dark goats, a native breed from Youyang County, Chongqing, China, have been raised for nearly a hundred years. They are characterized by unique phenotypic traits, such as dark skin, dark visible mucous membranes, and a white coat with a black mid-dorsal stripe [20,21]. Local White goats have white fur and skin all over their bodies. Youzhou Dark goats became a national genetic resource for livestock in 2009. They have developed excellent adaptability to the humid environmental conditions of Chongqing through long-term natural and artificial selection, significantly supporting local livestock production. While previous studies have addressed their nutritional values [22], the molecular mechanisms of their unique flavor remain unexplored. This study aims to reveal the molecular mechanisms of flavor formation in Youzhou Dark goats. By integrating flavoromics, metabolomics, and transcriptomics, we identified the key volatiles, metabolites, and genes in *Longissimus dorsi* muscle of Youzhou Dark (WY) and Local White (BY) goats. These findings providing a theoretical foundation for enhancing meat quality and breeding strategies for indigenous Chinese goat breeds.

## 2. Materials and Methods

### 2.1. Animals, Diets, and Feeding Management

All animal experimental procedures and protocols applied in this study were approved by the Animal Care and Ethics Committee of Chongqing Academy of Animal Sciences (Approval No: XKY-2020-B0614). In this study, ten 12-month-old clinically healthy castrated male Youzhou Dark (WY, N = 5) and Local White (BY, N = 5) goats were collected from Youyang County, Chongqing, China (geographic coordinates: 26°54′ N, 108°57′ E).

The experiment lasted 60 days, including 15 days for dietary adaptation and a 45-day feeding trial. The animals were fed twice (08:00 and 16:00) a day with feed intake ad libitum and with free access to drinking water. All goats were reared under identical feeding environments; the basal diet included concentrated feed and forage (peanut vines mixed). The composition and nutritional levels were in accordance with previous studies [22].

### 2.2. Slaughtering, Pretreatment, and Sample Collection

After the ten goats were slaughtered, the *Longissimus dorsi* muscles were collected, cleaned, and removal of excess fat and connective tissue. The longest back muscles were placed into 2 mL RNA-free freezing tubes. These tubes were immediately immersed in liquid nitrogen for rapid freezing preservation and subsequently stored at −80 °C for further analysis.

### 2.3. Volatile Compound Analysis

#### 2.3.1. Solid-Phase Microextraction (SPME) Conditions

Frozen goat muscle samples were retrieved from storage and thawed at 4 °C for 24 h. Each sample (5.0 g) was weighed, homogenized, and then transferred into in a headspace bottle. Volatile compounds in the sample were extracted using solid-phase microextraction (SPME) with a 1 cm fiber head. The SPME fiber was first incubated at 60 °C for 10 min, extracted for 40 min, and desorbed in the injection port of gas chromatography (GC) for 5 min.

#### 2.3.2. GC × GC Analysis Conditions

The GC × GC analysis was carried out using a gas chromatograph (7890A; Agilent Technologies Inc., Santa Clara, CA, USA) and a mass spectrometer (Pegasus 4D; LECO Inc., St. Joseph, MI, USA). First dimension column: A DB-WAX dimension (30 m × 250 μm× 0.25 μm) was used. The initial temperature maintained at 40 °C for 3 min, then increased to 250 °C at 5 °C/min and held for 5 min, and helium (99.9999%) 1.0 mL/min was injected without splitting. Second dimension column: A DB-17MS (2 m × 100 μm × 0.10 μm) was employed, with its temperature set 5 °C lower than that of the first dimension column. The temperature of the modulator was kept at 5 °C higher than that of the second column.

#### 2.3.3. Mass Spectrum Conditions

A LECO Pegasus BT 4D mass spectrometer (LECO, St. Joseph, MI, USA) was utilized for the detection of flavor substances. The transfer line and ion source temperature of time-of-flight mass spectrometry (TOF MS) were set at 250 °C. The acquisition frequency was 200 spectra/s. The mass spectrometer was operated in the electron ionization (EI) mode at 70 eV, using a range of *m*/*z* 35–550 and a detector voltage of 2036 V.

#### 2.3.4. Data Analysis

GC × GC-TOF-MS data analysis was performed using the Biodeep Platform analysis (Suzhou Panomix, Jiangsu, Suzhou, China). Multivariate statistical analyses, including principal component analysis (PCA), partial least squares discriminant analysis (PLS-DA), and orthogonal partial least squares discriminant analysis (OPLS-DA), were analyzed using the vegan package in R [23]. The differential metabolome profiles between the two goat groups were analyzed based on the OPLS-DA score plot. Differential metabolites were screened according to the difference between the variable importance in projection (VIP) and statistical significance (*p* < 0.05).

Principal Component Analysis (PCA) is widely used as a multivariate statistical analysis tool for analyzing sample variance, as it simplifies the data and highlights the interrelationships between distinct samples. PCA can reduce the dimensionality while preserving the maximal amount of variance, suppress random technical noise, and provide an unsupervised hypothesis-free visualization of innate sample clustering, outliers, and batch effects. These properties are essential in metabolomics data, where the number of variables (*m*/*z* features) greatly exceeds the number of biological replicates, and where unmodeled variance can propagate into downstream supervised analyses, leading to spurious biomarkers. By running PCA before supervised modeling, we (a) confirmed that the observed inter-group separation was not driven by latent batch variables, (b) identified and removed extreme spectral outliers that would otherwise inflate Type I error, and (c) determined the minimum number of latent components required for subsequent multivariate tests, thereby improving the model robustness and reproducibility [24].

#### 2.3.5. Flavoromics Analysis

The National Institute of Standards and Technology (NIST) database was used for the initial annotation of the detected volatile flavor compounds. The Odor and FlavorDB database was employed to annotate the detailed sensory flavor substances, including the flavor names, the minimum and maximum concentrations of flavor ranges, and sensory flavors [25,26]. Sankey diagrams and radar charts were generated using the ggplot package in R software, and a network diagram was drawn using Cytoscape software (v 3.8.2).

### 2.4. Untargeted Metabolomics Analysis

#### 2.4.1. Metabolites Extraction

First, *Longissimus dorsi* muscles (−80 °C) were lysed using a pre-cold (−20 °C) mixture of 75% methanol and 25% water. The lysed tissue was scraped and vortexed twice for 20 s. After centrifugation at 20,000× *g* and 4 °C for 30 min, the supernatants were collected and dried under nitrogen. Then, 200 µL of 50% acetonitrile solution was prepared with 2-chloro-l-phenylalanine to redissolve the sample, the supernatant was filtered using a 0.22 μm membrane, and it was transferred into the detection bottle for further analysis.

#### 2.4.2. Instrument Parameters

For liquid chromatography, metabolites were presented in positive and negative ion mode using an ACQUITY UPLC HSS T3 (2.1 × 100 mm, 1.8 µm) (Waters, Milford, MA, USA) with a column temperature of 40 °C. In positive polarity mode, mobile phase A was 0.1% formic acid in water, and mobile phase B was 0.1% formic acid in acetonitrile. In negative polarity mode, mobile phase A was 5 mM ammonium acetate, and mobile phase B was acetonitrile. The column was maintained at 40 °C. The flow rate and injection volume were set at 0.3 mL/min and 2 μL, respectively.

Mass spectrometric detection of metabolites was performed on an Orbitrap Exploris120 (Thermo Fisher Scientific, Waltham, MA, USA) with an ESI ion source and spray voltages of 3.5 and 2.5 kV, respectively. Sheath and auxiliary gases were set at 40 and 10 arbitrary units, respectively. The capillary temperature was 325 °C. The Orbitrap analyzer scanned over a mass range of *m*/*z* 100–1000 for the full scan at a mass resolution of 60,000 full-width at half-maximum height. Data-dependent acquisition (DDA) MS/MS experiments were performed with an HCD (High-Density-CD) scan. The normalized collision energy was 30 eV.

#### 2.4.3. Bioinformatics Analysis

Data processing involved database searching, and comparison was performed using the HMDB, massbank, LipidMaps, and mzcloud. Metabolite features were annotated by matching the accurately measured masses (±10 ppm) with the theoretical masses in the KEGG, HMDB, and METLIN databases. Additionally, an in-house SPI spectral database was also used for further annotation based on accurately measured masses and chromatographic retention times. The annotations from this database were used in MS/MS experiments by comparing the fragmentation spectra of samples and chemical standards.

### 2.5. Transcriptome Analysis

#### 2.5.1. RNA Extraction

Total RNA was extracted from muscle samples using TRIzol reagent (Invitrogen, Carlsbad, CA, USA) in accordance with the manufacturer’s instructions. RNA concentration and purity were determined using a NanoDrop 2000 spectrophotometer (Thermo Scientific, Waltham, MA, USA). RNA integrity was assessed using an Agilent Bioanalyzer 2100 system (Agilent Technologies, Palo Alto, CA, USA). Only RNA samples with an integrity number (RIN) ≥ 7.0 were selected for subsequent procedures.

#### 2.5.2. Sequencing

Briefly, mRNA was enriched from total RNA with Oligo (dT) beads. The enriched mRNA was fragmented into short fragments using fragmentation buffer and reverse transcribed into first-strand cDNA. Second-strand cDNA fragments (400–500 bp in length) were synthesized using DNA polymerase I, RNase H, deoxyribonucleotide triphosphates (dNTPs), and reaction buffer. The cDNA fragments were subjected to purification, end-repaired, A-tail-added, and ligated with sequencing adapters. The ligation products were verified via agarose gel electrophoresis, amplified via polymerase chain reaction (PCR), and finally sequenced using an Illumina NovaSeq 6000 (Illumina, San Diego, CA, USA).

#### 2.5.3. RNA-Seq Data Analysis

Raw reads were filtered to remove adapter-contaminated reads and low-quality reads. The resulting clean reads were mapped to the reference genome of domesticated goat (Capra_hircus.ARS1.cdna.all.fa) [27]. The expression levels of each gene were calculated using the fragments per kilobase of transcript per million mapped reads (FPKM) method. Differential expression gene (DEGs) analysis between the two goat groups was performed using DESeq2 software (v 1.20.0) [28]. A Q value < 0.05 and |log_2_(fold change)| ≥ 2 were used to define the differentially expressed genes (DEGs). Gene Ontology (GO) and Kyoto Encyclopedia of Genes and Genomes (KEGG) enrichment analyses of DEGs were conducted using the cluster Profile package of R software (v 3.2.0).

## 3. Results

### 3.1. Types of Flavor Substances Identification and Sensory Flavor Characteristics

#### 3.1.1. Identifying Types of Flavor Substances

**Figure 1:**
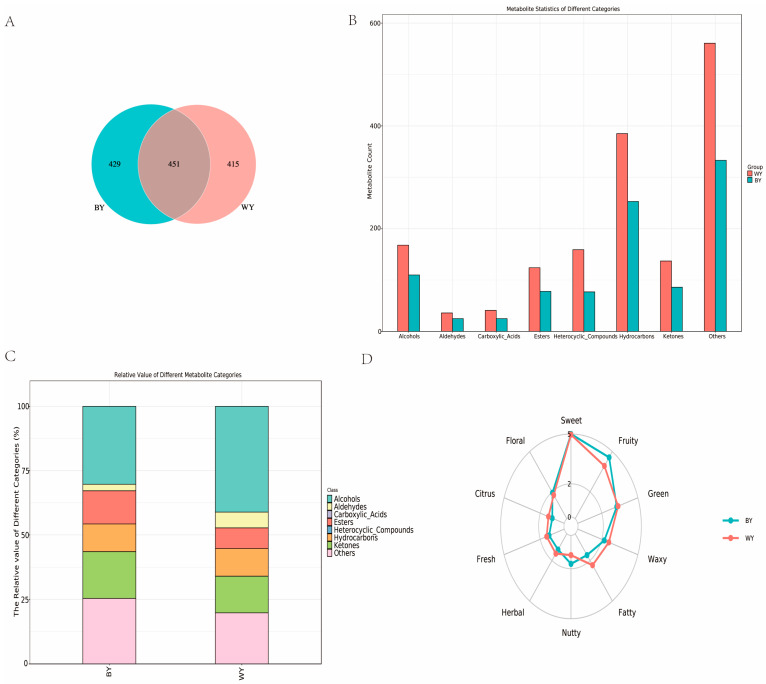
(**A**) Venn diagram that illustrates the overlap of volatiles from the two groups of mutton. (**B**) Number of volatile compounds in different categories. (**C**) Relative value of different volatile compounds. (**D**) Sensory flavor characterization with the designation in the outermost circle indicating the organoleptic flavor profile. The line indicates the detection frequency for the flavor substance (1–5, with a maximum of 5).

First, we investigated the differences in flavor compounds (*n* = 10) using GC × GC-TOF MS. A total of 1295 flavor compounds were identified using the NIST Mass Spectral Library (Supplementary Table S1). After annotating the detected substances and eliminating missing values, 880 volatiles were detected in BY goats, 866 in WY goats, and 451 shared between the two breeds (Figure 1A). The numbers of volatile compounds of the *Longissimus dorsi* muscle in WY and BY goats were as follows: hydrocarbons (186 vs. 152), alcohols (123 vs. 116), ketones (89 vs. 89), esters (67 vs. 93), aldehydes (38 vs. 19), heterocyclic compounds (25 vs. 32), carboxylic acids (13 vs. 23), and others (325 vs. 356) (Figure 1B). The relative contents of volatile compounds of the *Longissimus dorsi* muscle in WY and BY goats were as follows: alcohols (41.12% vs. 30.30%), ketones (14.21% vs. 18.14%), hydrocarbons (10.70% vs. 10.77%), esters (8.02% vs. 12.89%), aldehydes (6.16% vs. 2.53%), heterocyclic compounds (4.96% vs. 0.63%), carboxylic acids (0.24% vs. 1.28%), and others (15.59% vs. 23.44%) (Figure 1C).

#### 3.1.2. Analysis of Sensory Flavor Characteristics

The FlavorDB database was used to evaluate the sensory characteristics of the flavor compounds in the two goat samples (Figure 1D). Overall, the sensory flavor profiles of the WY and BY goats were similar. Fatty, citrus, fresh, herbal, and waxy aromas in WY goats were much higher than that in BY goats, and fruity and nutty aromas were much lower than that in BY goats. The two goat meats exhibited comparable sensory profiles for sweet and floral aromas.

### 3.2. Multivariate Statistical Analysis

#### 3.2.1. PCA of Volatile Compounds

PCA is widely used as a multivariate statistical analysis tool for analyzing sample variance, as it simplifies the data and highlights the interrelationships between distinct samples. In this study, PC1 accounted for 36.1% of the variability, and PC2 accounted for 12.2% (Figure S1A), indicating that the PCA results were statistically effective for characterizing sample differences.

#### 3.2.2. OPLS-DA of Volatile Compounds

OPLS-DA is a powerful approach for sample classification and the construction of discriminant models. It visualizes the extent of the difference between samples based on the correlation within data [29]. The OPLS-DA score plot demonstrated an independent separation of volatile compounds between the two goat meat groups (Figure S1B). The total variance in the contributions of the first two principal components was 46.6%. In the permutation test plots of the OPLS-DA models, all blue Q2 points were lower than the original blue Q2 points (Figure S1C), confirming that the established OPLS-DA model was precise and reliable.

### 3.3. Analysis of Differential Volatile Compounds

**Figure 2:**
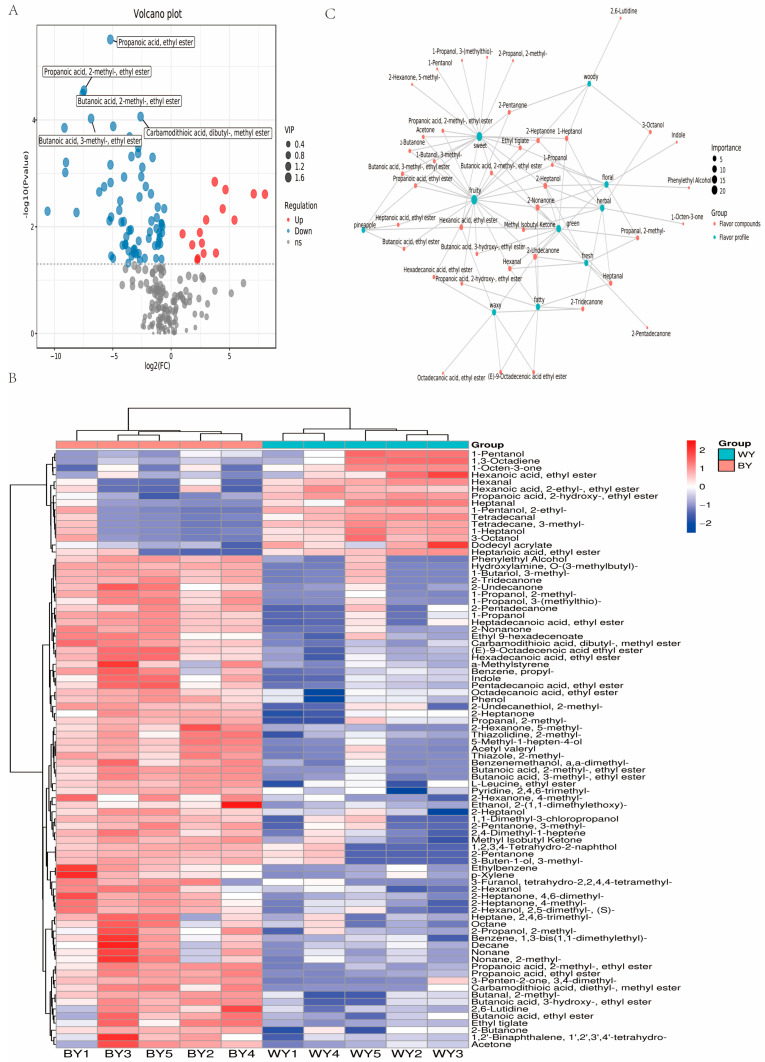
(**A**) The volcano plot for differential metabolites between different groups. (**B**) Heatmap of volatiles in the BY vs. WY goats. (**C**) Correlation network for the sensory flavor characteristics and flavor compounds. Compounds that showed significant differences between WY and BY goats are listed in Supplementary Table S2.

A total of 228 differential volatile compounds were identified in the current experiment. Using the screened criteria of *p*-value < 0.05 and VIP > 1, 85 volatile compounds showed significant differences between WY and BY goats (Supplementary Table S2). Of these, 15 were upregulated and 70 were downregulated (Figure 2A). These volatile compounds with differential expression contributed to the variations in the sensory flavor profiles of WY and BY goat meat. A heat map for the differential flavor volatiles revealed the good clustering of the samples (Figure 2B). After clustering, the flavor volatiles were divided into two groups exhibiting differentially expressed patterns between BY and WY goats. To explore the relationship between the sensory flavor characteristics and flavor compounds, a correlation network was established using the FlavorDB database. We selected the 10 most frequent flavors and 85 associated compounds in the diagram (Figure 2C). The top ten flavor profiles identified were sweet, fruity, woody, green, floral, fresh, fatty, wavy, herbal, and pineapple.

The relative odor activity value (ROAV) is a widely used indicator to evaluate the contribution of individual volatile component to the overall flavor [29]. Compounds with ROAV ≥ 1 are generally recognized as the main flavor substances in the samples [30]. All compounds identified in the meats of WY and BY goats, including the name, class, first dimension time, second dimension time, range of odor min, range of odor max, and odor character, are provided in Supplementary Table S2. In total, 73 compounds were identified with CAS annotations, including esters (16), alcohols (15), ketones (14), benzenoids (8), aldehydes (6), organoheterocyclic compounds (4), ethers (2), hydrocarbons (2), organohalogen compounds (3), organic oxygen compounds (1), phenylethyl alcohol (1), and other (1) (Supplementary Table S3).

### 3.4. Analysis of Differential Metabolites

**Figure 3:**
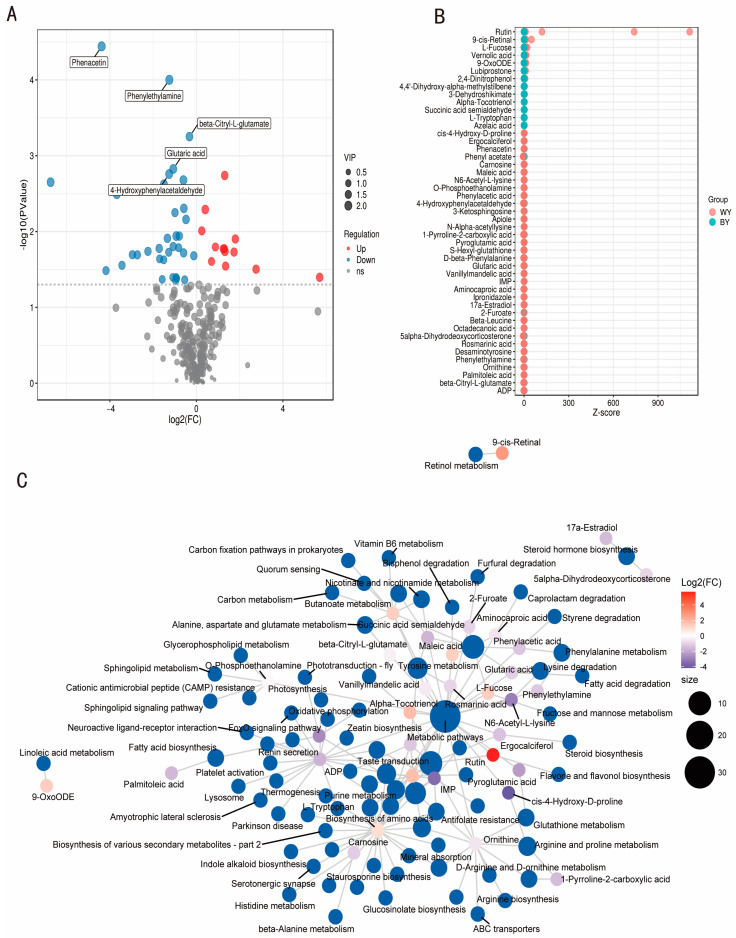
(**A**) The volcano plot for differential metabolites between different groups using untargeted metabolomics. (**B**) Z-score analysis overall trend and degree of difference in metabolite quantification values in BY vs. WY goats. (**C**) Correlation network for the 47 differential metabolites associated with different pathways. The metabolites that showed significant differences between WY and BY goats are listed in Supplementary Table S7.

Furthermore, untargeted metabolomics approaches were performed to identify differential metabolites that exhibited significant alterations between WY and BY goats. The results indicated that a total of 340 differential metabolites were detected in the present experiment. Using the screening thresholds of *p*-value < 0.05 and VIP > 1, 47 differential metabolites were significantly altered between WY and BY goats (Supplementary Table S7). Among these, 13 were found to be upregulated, and 34 were downregulated (Figure 3A). These differential metabolites were associated with the differences in sensory flavor profiles observed between the WY and BY goat meat. Z-score analysis visualized the overall trend and magnitude of metabolite differences. In the sample of WY goat meat, rutin had a significantly higher level (Figure 3B). Subsequently, KEGG pathway enrichment analysis was performed on the list of differential metabolites. Among the detected volatile compounds, esters, alcohols, ketones, benzenoids, and aldehydes were predominant. Notably, 2,3-butanedione, 2-methyl-butanal, heptanal, and 2-pentylfuran showed high VIP and ROAV values, indicating their major contribution to the characteristic aroma of goat meat (Figure 3C).

### 3.5. Identification of Differential Genes and Functional Enrichment Analysis

#### 3.5.1. Identification of DEGs

To gain deeper insights into the differences in gene expression patterns between different goat breeds, comparative RNA sequencing (RNA-seq) was applied to analyze the muscle transcriptome. PCA revealed distinct principal components between two goat breeds: PC1 explained 33% of the total variability, and PC2 accounted for 23%. The distances in WY were larger than those in BY goats (Figure S2A). A total of 96 genes with significant differences were identified between the BY and WY groups, including 79 upregulated genes and 17 downregulated genes. Different DEG gene expression patterns were observed between BY and WY goats, and the DEGs shared by the two groups were divided into nine distinct clusters. The gene expression profiles of BY and WY goats were significantly different (Figure S2B). The expression of DEGs in the two groups is displayed in Figure S2C.

#### 3.5.2. Functional Enrichment Analysis

**Figure 4:**
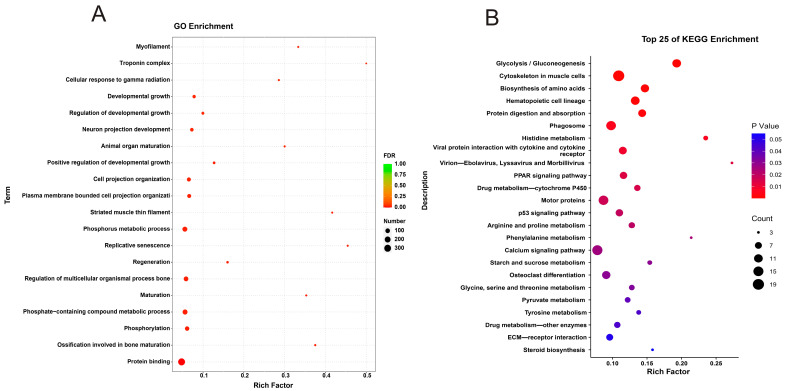
(**A**) GO and (**B**) KEGG enrichment analysis of transcriptomics analysis in *longissimus dorsi* muscle of the two types of goat meat. The top 20 GO and top 25 KEGG pathways are presented from low to high, according to the Padj.

GO enrichment analysis was conducted to explore the functional significance of the DEGs in the BY and WY groups, yielding 6429 terms in total. DEGs were classified into three categories: biological processes (BPs), molecular functions (MFs), and cellular components (CCs). The top ten enriched terms for each category were selected for visualization. DEGs were mainly enriched regarding phosphorus metabolic processes, regulation of multicellular organismal processes, phosphate-containing compound metabolic processes, and protein binding (Figure 4A, Supplementary Table S4). KEGG enrichment analysis was performed, and the DEGs were mapped to 287 distinct pathways (Figure 4B, Supplementary Table S5). Key enriched pathways included the peroxisome proliferator-activated receptor (PPAR) signaling pathway, glycine, serine and threonine metabolisms, and arginine and proline metabolisms. Based on these results, we hypothesized that these pathways and their associated genes might be the potential functional roles and pathways linked to the mutton flavor in the BY and WY groups.

**Figure 5:**
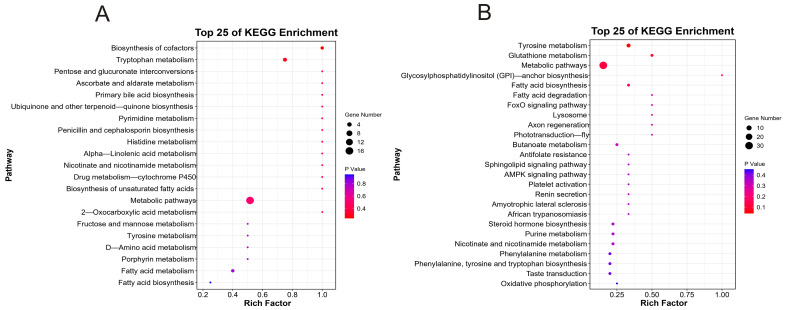
KEGG enrichment analysis of transcriptomics analysis in *longissimus dorsi* muscle of the two types of goat meat. The top 25 KEGG pathways according to (**A**) flavor enrichment and (**B**) flavor-related metabolites.

The top 25 KEGG enrichment pathways related to flavor formation in goat meat were identified to determine the relationship of the metabolic pathways linked with flavor formation (Figure 5A). The top five pathways were glycolysis, cytoskeleton in muscle cells, biosynthesis of amino acids, hematopoietic cell lineage, and protein digestion and absorption. These pathways are crucial for understanding the biochemical processes that contribute to the flavor of goat meat. These pathways encompass a range of metabolic processes, including lipid, amino acid, and cofactor biosynthesis, metabolic pathways, and tryptophan metabolism. These findings provide valuable insights into the biochemical mechanisms underlying the formation of flavor compounds in goat meat.

Furthermore, KEGG enrichment analysis was carried out to reveal the metabolic and signaling pathways associated with flavor formation (Figure 5B). These results identified 25 pathways related to flavor formation based on the DEGs. The DEGs were mainly enriched in metabolic pathways related to lipid and amino acid metabolism and were associated with various metabolic pathways that play crucial roles in flavor formation. For example, the tyrosine metabolism pathway is involved in synthesizing phenolic compounds that contribute to the characteristic flavor of goat meat. The glutathione metabolic pathway is important for maintaining oxidative stability and preventing the formation of off-flavors. The PPAR signaling pathway is involved in the regulation of fatty acid metabolism and production of flavor compounds. These results suggest that the differences in flavor compounds between the two goat breeds may be attributed to the differential expression of genes involved in these metabolic pathways.

### 3.6. Analysis of the Tyrosine, Phenylalanine, and Tryptophan Metabolism Pathways

**Figure 6:**
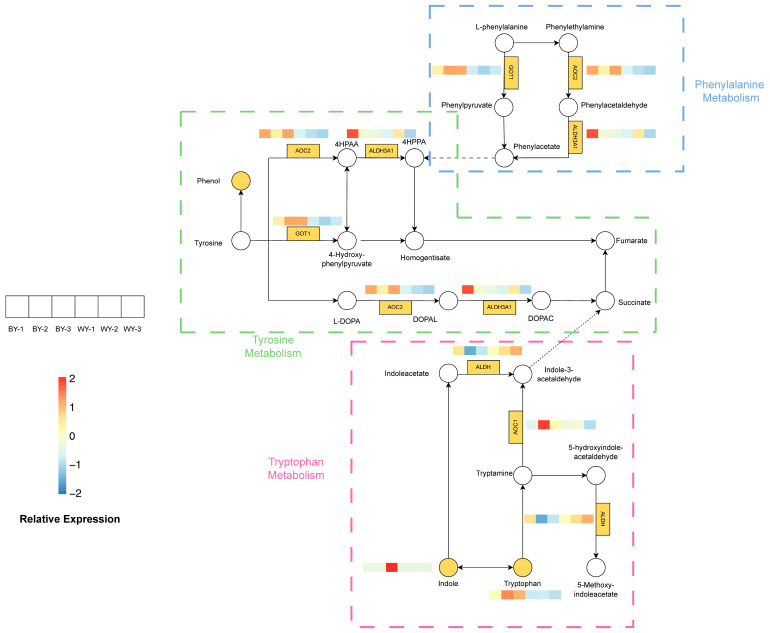
DEGs and metabolites in the tyrosine, phenylalanine, and tryptophan metabolism pathways network in the BY and WY groups play significant roles in synthesizing volatile compounds.

To further illustrate the connection between volatile compounds and DEGs, we focused on important differentially expressed metabolites and DEGs in a few key pathways and constructed the pathway network (Figure 6). Among these pathways, the tyrosine, phenylalanine, and tryptophan metabolic pathways are directly related to synthesizing flavor compounds. Tyrosine metabolism is involved in producing phenolic compounds that contribute to the characteristic flavor of goat meat. Phenylalanine is an aromatic amino acid that serves as a precursor of various flavor compounds. Tryptophan metabolism leads to the formation of various nitrogen-containing compounds that affect the aroma and taste of the meat.

The tyrosine, phenylalanine, and tryptophan metabolic pathways were found to play significant roles in the meat of the BY and WY groups. Specifically, the tyrosine metabolism pathway contributes to the synthesis of phenolic compounds, which are characteristic of the goat meat flavor. The glutathione metabolic pathway is essential for maintaining oxidative stability and preventing off-flavor formation. Differences in flavor compounds between the two goat breeds may be linked to the differential expression of genes involved in these metabolic pathways. At the gene level, the levels of *AOC2*, *ALDH3A1*, *GOT1* and *ALDH*, which are involved in tyrosine and phenylalanine metabolism, were higher in the BY group. Expression of *ALDH*, involved in tryptophan metabolism, was higher in the WY group (Figure 6). The relative expression levels of these metabolites and their associated genes were analyzed to understand their contributions to the distinct flavor profiles of BY and WY goat meat.

## 4. Discussion

Mutton is an important source of high-quality protein, characterized by low cholesterol and fat content. It boasts a tender texture and high digestibility, making it increasingly popular among consumers. In China, mutton consumption has gradually increased in recent years. Meat flavor is a pivotal factor that influences meat palatability and consumer acceptance. When comparing different quality attributes of meat products, volatile flavor compounds offer a more comprehensive basis for differentiation than taste or texture [31]. These volatile substances in meat are primarily generated through biochemical processes, including lipid oxidation, the Maillard reaction, and thiamin breakdown, and they mainly fall into categories such as alcohols, aldehydes, esters, ketones, hydrocarbons, and furans [32]. Among the detected volatile compounds, esters, alcohols, ketones, benzenoids, and aldehydes were predominant. Notably, 2,3-butanedione, 2-methyl–butanal, heptanal, and 2-pentyl–furan, showed high VIP and ROAV values, indicating their major contribution to the characteristic aroma of goat meat. The diversity of flavor compounds present in these goat breeds may account for their complex flavor profiles.

Volatiles in meat are mainly alcohols produced by the oxidation of linoleic acid degradation products, most of which have pleasant odors and can enhance the overall flavor of meat [5]. Alcohols also account for a high proportion of the volatile flavor compounds in meat. 1-octen-3-ol is an important alcohol-flavored substance in goat meat, characterized by mushroom-like aroma characteristics [33]. The content of 1-octen-3-ol in WY goats was significantly higher than that in BY goats, which is consistent with earlier findings related to sheep [34]. This compound is therefore likely to contribute substantially to the flavor development of goat meat. Alcohols typically exhibit a range of odor profiles, such as plant-like, rancid, or chemical notes. Previous research has shown that increased levels of alcohols, particularly unsaturated alcohols, could impact the flavor of lamb meat, whereas other types of alcohols have no notable effect [30]. Although the alcohol threshold in goat meat was relatively low, it could weakly affect the meat flavor. Goats themselves have high sensory detection thresholds, thus synergistically affecting the overall aroma of goat meat.

Aldehydes represent the main volatile compounds in pork, chicken, and sheep meat [5,12,35]. Here, the content of heptanal in WY was significantly higher than that in BY goats, and the ROAV values of heptanal were 16.39 in WY and 0.55 in BY goats. This notable difference indicates that heptanal can serve as a reliable indicator to distinguish between the WY and BY groups. Most aldehydes possess fatty aromas and act as crucial intermediates in Maillard or lipid oxidation reactions, participating in the interactions between amino acids and carbonyl groups. Prior research has shown that the heptanal content was higher in the volatile compounds of Dorper sheep than in those of Tan and Hu sheep [34]. This discrepancy suggests that the fatty acid oxidation pathways may differ between sheep and goats, and variations in heptanal levels could further contribute to the distinct flavor profiles of these species.

Ketones in mutton are also produced via the thermal oxidation or degradation of UFAs or amino acids. These compounds typically have a fruity, buttery, and creamy flavors, which exert a positive influence on the overall flavor of meat [35]. 2,3-butanedione showed the highest concentration in the *longissimus dorsi* muscle of WY goats and exhibited mainly sweet mushroom flavor-distinct characteristics. Its content in WY was significantly higher than in BY goats; the ROAV values of 2,3-butanedione were 100 in WY and 87.62 in BY goats. Notably, the ROAV values of ketones were far higher than those of aldehydes, confirming that ketones positively contributed to the volatile flavor of goat meat. 2-pentanone was also detected in WY goat mutton. As documented in previous research, 2-pentanone is a marker of product spoilage that contributes to the flavor changes in raw meat and meat products [13,36]. The concentration of 2-heptanone was higher in BY than in WY goats. Although ketones in goat meat exhibit a lower ROAV value compared with aldehydes, they undergo coordinated formation during the process of meat flavor. They play an integral part in shaping the final sensory quality of goat meat.

In cooked goat, 2-pentyl–furan in meat has musty and floral odors [37]. This compound was found in high concentrations in the muscle of WY goats and exhibited distinct flavor characteristics, primarily notes of vegetables and green beans. Furan compounds are produced through multiple pathways, including the Maillard reaction, lipid thermal degradation, and the interaction between these two reactions, which are recognized as the main processes producing flavor and aroma compounds [38,39]. Given its abundance and distinct sensory attributes, 2-pentyl–furan also qualifies as a critical flavor-active and odor-active compound, playing a key role in shaping the overall flavor profile of the two goat meat samples.

Untargeted metabolomics showed rutin was significantly higher level in the sample of the WY group, and the different level of metabolites between WY and BY goats was closely associated with metabolic pathways, especially tyrosine metabolism. Rutin influences tyrosine and tryptophan metabolism (Figure 3). For example, rutin prevents UV-induced oxidative modifications of tyrosine and tryptophan derivatives in fibroblasts. It significantly reduces the levels of 3Cl-tyrosine, a UV-induced oxidative product of tyrosine, and increases tryptophan levels by approximately 10%. Additionally, rutin may affect tyrosine kinase activity. Studies have shown that rutin inhibited the tyrosine kinase activity of the epidermal growth factor receptor (EGFR), thereby suppressing EGFR-mediated signaling pathways and exerting anticancer effects. The tyrosine metabolism pathway primarily describes the catabolism and transformation of tyrosine into various biologically important molecules. Tyrosine metabolizes to produce hormones such as thyroxine and triiodothyronine, neurotransmitters such as L-DOPA, dopamine, adrenaline, or noradrenaline, and serves as a precursor for melanin and Coenzyme Q10.

In this study on flavor formation in goat meat, the tyrosine and tryptophan metabolic pathways were found to play significant roles. The tyrosine metabolism pathway contributes to the synthesis of phenolic compounds, which are characteristic of goat meat flavor. The glutathione metabolic pathway is essential for maintaining oxidative stability and preventing off-flavor formation. The PPAR signaling pathway is involved in the regulation of fatty acid metabolism and flavor compound production. The differences in flavor compounds between the two goat breeds may be linked to the differential expression of genes involved in these metabolic pathways (Figure 6). For instance, in the tyrosine metabolism pathway, phenylacetate and homogentisate are intermediate metabolites that can influence the flavor profile. Indole-3-acetaldehyde and 5-hydroxyindole–acetaldehyde are key intermediates in the tryptophan metabolic pathway that contribute to flavor characteristics. The relative expression levels of these metabolites and their associated genes were analyzed to understand their contributions to the distinct flavor profiles of BY and WY goat meat.

## 5. Conclusions

This study combined flavoromics, metabolomics, and transcriptomics to investigate the flavor differences between Youzhou Dark and Local White goats. Eighty-five volatile compounds differed significantly between the breeds, and key markers such as heptanal, 1-octen-3-one, 2,3-butanedione, 2-methyl–butanal, and 2-pentyl–furan effectively distinguished them. Genes involved in tyrosine and phenylalanine metabolism (AOC2, ALDH3A1, GOT1, ALDH) may contribute to these differences. Overall, these findings enhance the understanding of flavor formation mechanisms in native Chinese goats and can inform breeding and meat quality improvement strategies.

## Data Availability

The original contributions presented in this study are included in the article/Supplementary Material. Further inquiries can be directed to the corresponding authors.

## Supplementary Materials

The following supporting information can be downloaded from https://www.mdpi.com/article/10.3390/foods14234114/s1: Figure S1. PCA score chart for volatile compounds. OPLS-DA score chart for volatile compounds. The permutation test OPLS-DA model for volatile compounds. Figure S2. PCA analysis of the two types of goat meat. All DEGs were divided into nine clusters according to their expression levels. Heatmap of the expression of the DEGs in common. Table S1. The list of flavor compounds. Table S2. The list of differential volatile compounds. Table S3. The list of relative odor activity value (ROAV). Table S4. The list of differentially expressed genes. Table S5. The list of GO enrichment. Table S6. The list of KEGG pathway enrichment. Table S7. The list of differential metabolites.
