# Supplementary material for: Characterization of Mutton Volatile Compounds in Youzhou Dark Goats and Local White Goats Using Flavoromics, Metabolomics, and Transcriptomics
Source: Foods. 2025 Dec 1;14(23):4114. doi: 10.3390/foods14234114 (PMC12692560; doi:10.3390/foods14234114)
Supplement: Supplementary file 1 [file foods-14-04114-s001.zip › foods-3958814-supplementary.docx.author revise.pdf]

Supporting information

Characterization of Mutton Volatile Compounds in Youzhou Dark Goats and Local White Goats Using Flavoromics, Metabolomics and Transcriptomics

Jie Li, Shipeng Lv, Cancan Chen, Jing Jiang, Xiaoyan Sun, Gaofu Wang*, Hangxing Ren*
Chongqing Academy of Animal Sciences, Rongchang 402460, China;

***Correspondence**: Gaofu Wang, wanggaofs20031216@163.com; Hangxing Ren, rhxe@163.com

Supplementary figures:

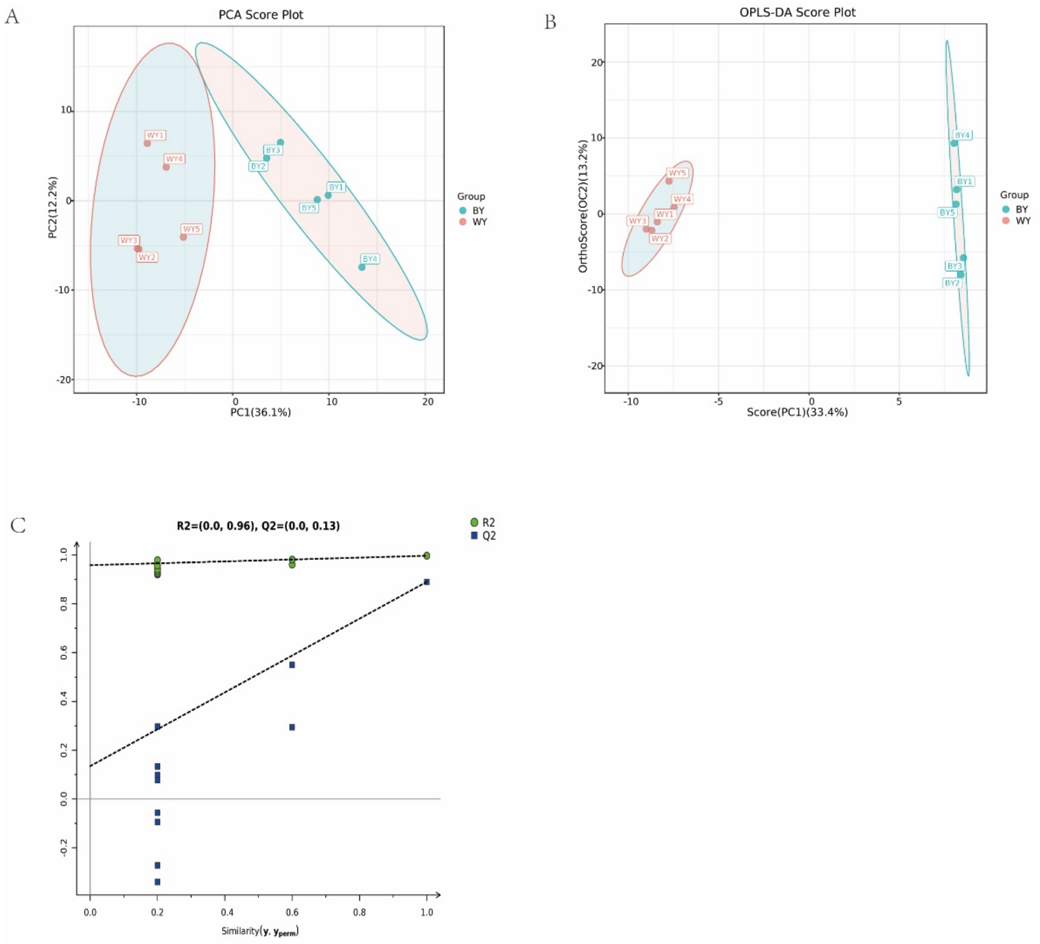

Figure S1. (A) PCA score chart for volatile compounds. (B) OPLS-DA score chart for volatile compounds. (C) The permutation test OPLS-DA model for volatile compounds.

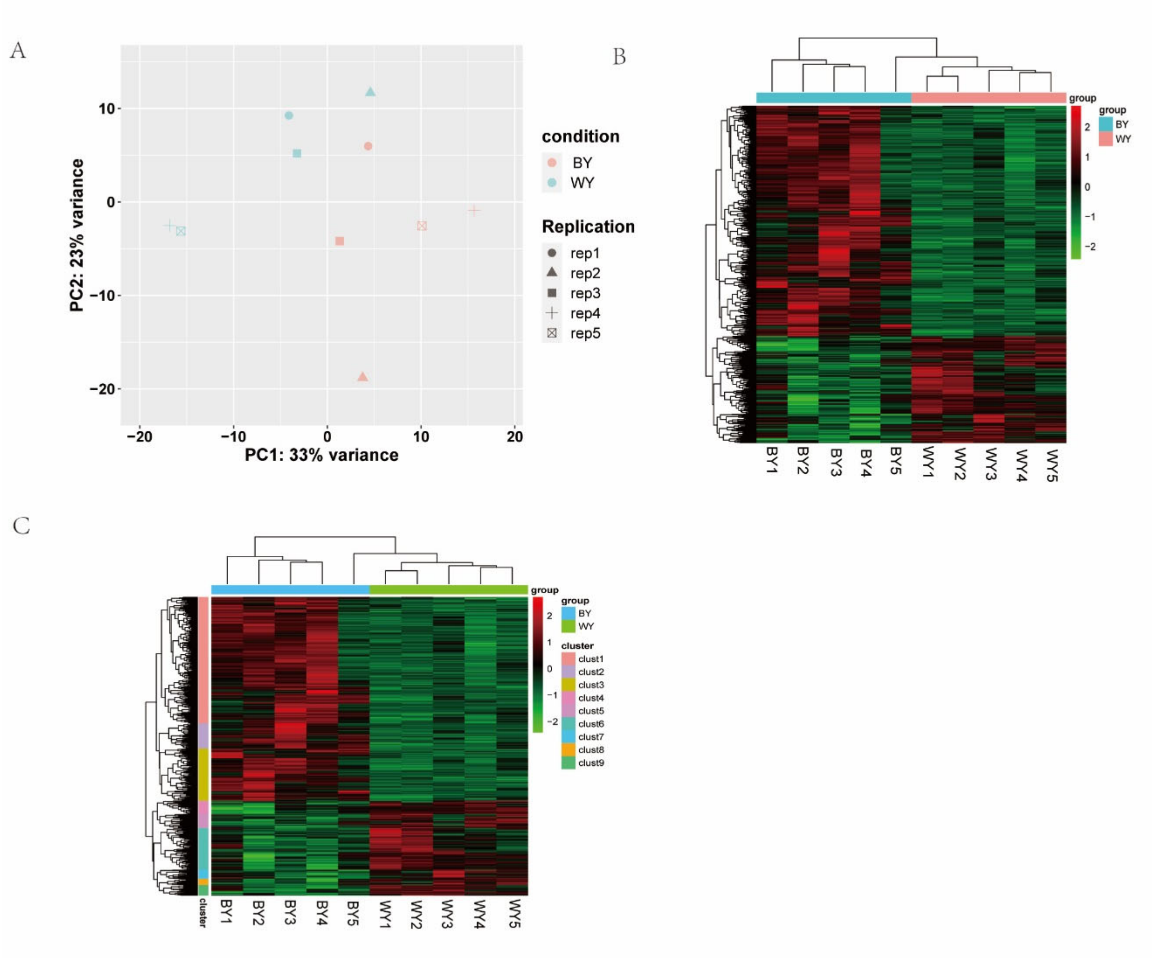

Figure S2. (A) PCA analysis of the two types of goat meat. (B) All DEGs were divided into nine clusters according to their expression levels. (C) Heatmap of the expression of DEGs common.
